# Advancing breast cancer biomarkers: a centromere-related gene signature integrated with single-cell analysis for prognostic prediction

**DOI:** 10.3389/fimmu.2025.1678603

**Published:** 2025-12-04

**Authors:** Ye Lu, Shengbin Pei, Wenxiang Zhang, Zheng Qu, Xiangyu Wang, Siqing Liu, Hao Dong, Kan Yonemori, Yi Fang, Xiangyi Kong, Jing Wang, Jidong Gao

**Affiliations:** 1 Department of Breast Surgical Oncology, National Cancer Center/National Clinical Research Center for Cancer/Cancer Hospital, Chinese Academy of Medical Sciences and Peking Union Medical College, Beijing, China; 2 Department of Breast Surgical Oncology, National Cancer Center/National Clinical Research Center for Cancer/Cancer Hospital & Shenzhen Hospital, Chinese Academy of Medical Sciences and Peking Union Medical College, Shenzhen, China; 3 Department of Oncoplastic Reconstructive and Aesthetic Breast Surgery, Plastic Surgery Hospital, Chinese Academy of Medical Sciences and Peking Union Medical College, Beijing, China; 4 Department of Medical Oncology, National Cancer Center Hospital, Tokyo, Japan

**Keywords:** breast cancer, CENPs, MMP1, prognostic model, single-cell RNA sequencing

## Abstract

**Background:**

Breast cancer (BC) is the most common malignancy among women and shows significant heterogeneity in its prognosis. Among the subtypes, triple-negative breast cancer (TNBC) has the poorest prognosis. Despite advancements in molecular stratification tools, such as Oncotype DX and MammaPrint, prognostic models based on chromosomal instability are still insufficient. The centromere protein (CENP) family, which plays a crucial role in maintaining genomic stability, is associated with tumor progression due to aberrant expression.

**Methods:**

In this study, we integrated multi-omics data, including RNA transcriptomic profiles and single-cell RNA sequencing, to identify gene modules linked to CENPA using weighted gene co-expression network analysis (WGCNA). We developed a prognostic model employing Cox regression and the LASSO algorithm. Validation was performed on independent cohorts, and the model's performance was tested by stratifying patients into high- and low-risk groups based on their five-year survival rates (p < 0.001).

**Results:**

The prognostic model effectively identified high- and low-risk patient groups, with the high-risk group showing significantly reduced five-year survival. Single-cell analysis revealed that CENPA-high subpopulations were enriched in proliferative tumor cells and were associated with an immunosuppressive tumor microenvironment.

**Conclusion:**

This study is the first to establish a CENP-based prognostic model for BC, offering novel biomarkers and potential therapeutic targets for personalized treatment. Additionally, the biological function of the key molecule MMP1 was validated through both *in vitro* and *in vivo* experiments.

## 1 Introduction

Breast cancer is one of the most common malignant tumors affecting women’s health worldwide, with approximately 2.3 million new cases and 670, 000 deaths reported in 2025, making it the second most common cancer after lung cancer, and the fourth leading cause of cancer-related deaths (1–3). The disease is characterized by high heterogeneity, with distinct molecular subtypes exhibiting markedly different clinical behaviors and prognoses, thus posing considerable challenges for precision treatment (4, 5). Although patients with early-stage BC generally have favorable prognoses, metastasis remains the primary cause of treatment failure and mortality (6, 7). Studies have shown that about 3% to 10% of patients present with distant metastasis at initial diagnosis, and among those diagnosed at early stages, 30% to 40% may eventually develop metastases, with a five-year survival rate of only around 20% (8). It is crucial to clarify the molecular mechanisms that drive BC metastasis and discover new biomarkers for early detection and treatment, aiming to enhance patient survival and outcomes (1, 4, 9). Traditional clinicopathological indicators, such as TNM staging and hormone receptor status, have limited predictive accuracy for individualized survival outcomes, particularly in aggressive subtypes like TNBC (10–12). Consequently, there is an urgent need to develop more stable and reliable molecular classification tools and prognostic models, which have become a significant focus of BC research (13, 14).

Aneuploidy, characterized by abnormal chromosome numbers, is common in malignancies and is associated with the gain or loss of specific oncogenes and tumor suppressor genes, thereby contributing to tumorigenesis, malignant progression, metastasis, and poor prognosis (15). Identifying key molecular drivers of aneuploidy may facilitate the development of targeted therapies aimed at this process (16). The centromere, a crucial chromatin structure during mitosis and meiosis, serves as a platform for kinetochore assembly (17). At the molecular level, centromeres comprise highly repetitive DNA sequences and conserved binding proteins collectively known as centromere proteins (CENPs). During cell division, CENPs assemble to form the kinetochore, bind microtubules, and orchestrate accurate chromosome segregation, which is essential in maintaining genomic stability (18, 19). In recent years, multiple studies have revealed the significant role of CENPs in cancer development and progression (20). Basic research has shown that CENPs rapidly localize to DNA double-strand break (DSB) sites following radiation-induced damage, implicating them in DNA damage response and repair. Ding et al. demonstrated that the MBNL1-AS1/ZFP36/CENPA axis downregulates CENPA expression by reducing mRNA stability, thereby inhibiting proliferation and cancer stem-like properties in BC cells, suggesting that CENPs may serve as promising therapeutic targets (21, 22).

To better assess prognosis in BC patients, a range of molecular feature–based prognostic models have been developed (23, 24). Clinically validated multigene assays, including the 21-gene recurrence score (Oncotype DX) and the 70-gene signature (MammaPrint), have proven the value of incorporating molecular biology indicators into outcome prediction, with the 21-gene recurrence score helping predict recurrence risk and guide chemotherapy decisions, and the 70-gene signature aiding in prognosis assessment and personalized treatment selection. These models primarily consist of genes involved in proliferation and cell cycle regulation (25, 26). However, beyond uncontrolled proliferation, mitotic errors and chromosomal instability are also fundamental contributors to tumor progression. This suggests that genes regulating mitosis and chromosome segregation should be considered essential components of prognostic models (27). The CENP gene family has attracted increasing interest owing to its central role in chromosomal stability. Several CENP genes, including CENPA, CENPF, and CENPU, are dysregulated in BC and are associated with patient prognosis, underscoring their relevance in tumor biology (28–31). Nonetheless, a prognostic model centered specifically on centromere proteins has not yet been developed, and the potential predictive value of CENPs remains to be fully explored.

Based on this context, the present study aimed to construct and validate a BC prognostic model based on CENP-related genes to enhance the prediction of patient prognosis. We integrated multi-omics data, including RNA-seq data from BC tissues to capture global transcriptomic profiles, and single-cell RNA sequencing data to analyze gene expression at the cellular level within tumors, providing a multidimensional perspective on the CENPA-associated molecular network. We employed WGCNA to identify gene modules closely related to CENPA. Prognostic genes were further refined using Cox proportional hazards regression and the LASSO method to construct a risk signature. The model was subsequently validated in independent BC cohorts by comparing survival outcomes across risk subgroups. This CENPA-focused approach, combining multi-omics and multiple analytical methods, aims to develop a robust and reliable prognostic model to support prognosis evaluation and guide individualized treatment strategies in BC.

## 2 Method

### 2.1 Data acquisition and preprocessing

RNA sequencing data and clinical information for BC patients were retrieved from TCGA, including 1, 118 tumor and 113 normal samples. Only samples with complete clinical data and high-quality RNA-seq data (≤10% missing values) were included; others with poor data quality were excluded. Additionally, transcriptomic and clinical data (GSE20685) were obtained from the GEO database, and single-cell data (GSE174588) from 22 samples (11 BC, 11 normal). GEO samples were included if they had clinical data, a BC diagnosis, and met quality control standards. Three hundred ninety-five centromere-related genes were identified from the Genecards database, and only those with a correlation coefficient > 0.2 were analyzed further.

### 2.2 Single-cell analysis

To ensure high-quality scRNA-seq data from 11 BC samples, we performed rigorous preprocessing and quality control using the “Seurat” and “SingleR” R packages. First, cells were filtered based on the following quality control criteria: genes needed to be expressed in at least three cells, and each cell had to express a minimum of 50 genes. Cells with fewer than 50 genes or high mitochondrial gene content (above 5%) were excluded to avoid low-quality or stressed cells. We further filtered cells with a high proportion of genes expressed from mitochondrial DNA, indicative of potential cell damage or stress. Next, we used the “NormalizeData” function with a scaling factor of 10, 000 to normalize the gene expression data. To identify genes with the most expression variability across cells, we applied the “FindVariableFeatures” function using the variance-stabilizing transformation (vst) method, selecting the top 1, 500 most variable genes for downstream analysis. Cell type annotation was performed using the “SingleR” method, which assigns cell identities based on reference datasets like the Human Primary Cell Atlas. Finally, we used the “Monocle” package to investigate cell differentiation trajectories and perform differential gene analysis to understand the gene expression dynamics in the BC samples.

### 2.3 Consensus clustering analysis

To gain insights into gene expression patterns associated with cell differentiation trajectories, we utilized the K-means clustering algorithm from the “ConsensusClusterPlus” package to analyze differentially expressed genes (32). We set the maximum number of clusters to 9 (max K = 9) and identified the optimal cluster count as two by examining the consistency matrix of the clustering outcomes. Additional analyses were performed to investigate survival variations among the clusters, examine heterogeneity within the tumor microenvironment, and evaluate the differences in immune cell infiltration and the expression of immune checkpoints.

### 2.4 Construction of prognostic model

To investigate differentiation trajectories in BC and identify gene modules associated with clinical features, we extracted expression data for differentially expressed genes from the TCGA database and performed weighted gene co-expression network analysis (WGCNA). First, the optimal soft-thresholding power (β) was determined using the pickSoftThreshold function to ensure the network approximated a scale-free topology (R² ≥ 0.9). Gene modules were then identified using hierarchical clustering and dynamic tree-cutting, with a minimum module size set to 30 genes. To assess module stability, we performed bootstrapping with 100 iterations to evaluate the robustness of the modules and their correlation with clinical traits. Finally, genes exhibiting expression differences between BC and normal tissues were selected and cross-referenced with a gene set related to centromere proteins to identify critical genes.

### 2.5 Construction of nomograms

Through univariate and multivariate Cox regression analyses, we explored the relationship between age, sex, TMN staging, and risk scores with the survival time of BC patients. We used the “rms” and “regplot” packages in R to create nomograms that integrate age, sex, TMN staging, and risk scores to predict the survival duration of BC patients.

### 2.6 Immune cell infiltration analysis

We calculated the proportion of immune infiltrating cells using the “CIBERSORT” algorithm and visualized the correlations between different immune cell types and three variables (MMP1, TFPI, and risk score) through heatmaps.

### 2.7 Chemosensitivity analysis

Using the “oncoPredict” package, we calculated the IC50 values of various chemotherapy drugs for different risk groups of patients to assess their chemosensitivity. Wilcoxon tests were employed to analyze differences between the various subtypes.

### 2.8 Candidate drug prediction and molecular docking

The two-dimensional structures of the chosen drugs were retrieved from the PubChem website and subsequently imported into “Chem3D” software for conversion into three-dimensional models. Protein structure information for the genes was sourced from the PDB database. We utilized “PyMol” software to eliminate water molecules and small ligand entities from the receptor structures.

### 2.9 Cell culture and tissue collection

Tissue samples were obtained from the Chinese Academy of Medical Sciences Cancer Hospital and stored at -80 °C. Between February 2023 and November 2024, we collected 10 pairs of samples, consisting of tumor tissue (T) and adjacent normal tissue (N), from patients with BC who underwent tumor resection. The Cancer Hospital of the Chinese Academy of Medical Sciences ethics committee approved the study (Approval No. 2010-SR-091). The BC cell lines MDA-MB-231 and HCC1806 were acquired from the Shanghai Institute of Life Sciences Cell Resource Center. These cell lines were maintained in DMEM medium (Gibco BRL, USA) at 37 °C with 5% CO2 and supplemented with 10% fetal bovine serum (FBS) from Gibco BRL, USA.

### 2.10 MMP1 knockdown and transcription

Targeted siRNA against MMP1 and corresponding negative control (Si-NC) were designed by RiboBio (Guangzhou, China). Cell transfection was performed using Lipofectamine 3000 (Invitrogen), and transfection efficiency was verified using RT-qPCR. qRT-PCR utilized SYBR Green kits (Vazyme, Nanjing, China), with GAPDH as the internal control for quantification. All primers were designed by QinKe Biotechnology (Beijing, China), and specific sequences are provided in **Supplementary Table 1**.

### 2.11 Functional experiments post-MMP1 knockdown

Cell proliferation was assessed using the CCK-8 method: 2×10³ cells per well were seeded in 96-well plates, and 10 μl of CCK-8 reagent (Vazyme) was added to each well and incubated in the dark for 2 hours. Absorbance was measured at 450 nm using a microplate reader (Thermo) at various time points (0–120 hours). For colony formation assays, 1, 000 transfected cells were cultured for 14 days, fixed with 4% paraformaldehyde, and stained with crystal violet (Solarbio) for counting colonies. Transwell assays were conducted to evaluate cell migration and invasion: 2×10^4^ cells were seeded in the upper chamber with serum-free media (Matrigel was pre-coated in invasion assays), and 10% serum media was added to the lower chamber. After 24 hours, the cells were fixed and stained, and invading cells were counted under a microscope. All experiments were performed in triplicate.

### 2.12 Animal models

The Animal Experiment Ethics Committee of Nanjing Medical University approved all animal experiments in this study. For the xenograft model, we utilized 5-week-old female BALB/c mice. Stable MDA-MB-231 cells, either transfected with siRNA or negative controls (Si-NC), were implanted into the mice’s bilateral groin for tumor development assessments. Tumor weight and volume measurements were taken every 5 days. After 20 days, the xenograft tumors and adjacent tissues were surgically removed and weighed.

### 2.13 Statistical analysis

The experiment data were evaluated with GraphPad Prism (version 8.0). The results from three separate experiments are shown as means ± standard deviation (SD). Group differences were analyzed using the Student’s t-test.

## 3 Results

### 3.1 Analysis of single-cell sequencing data and cell differentiation trajectories

**Figure 1:**
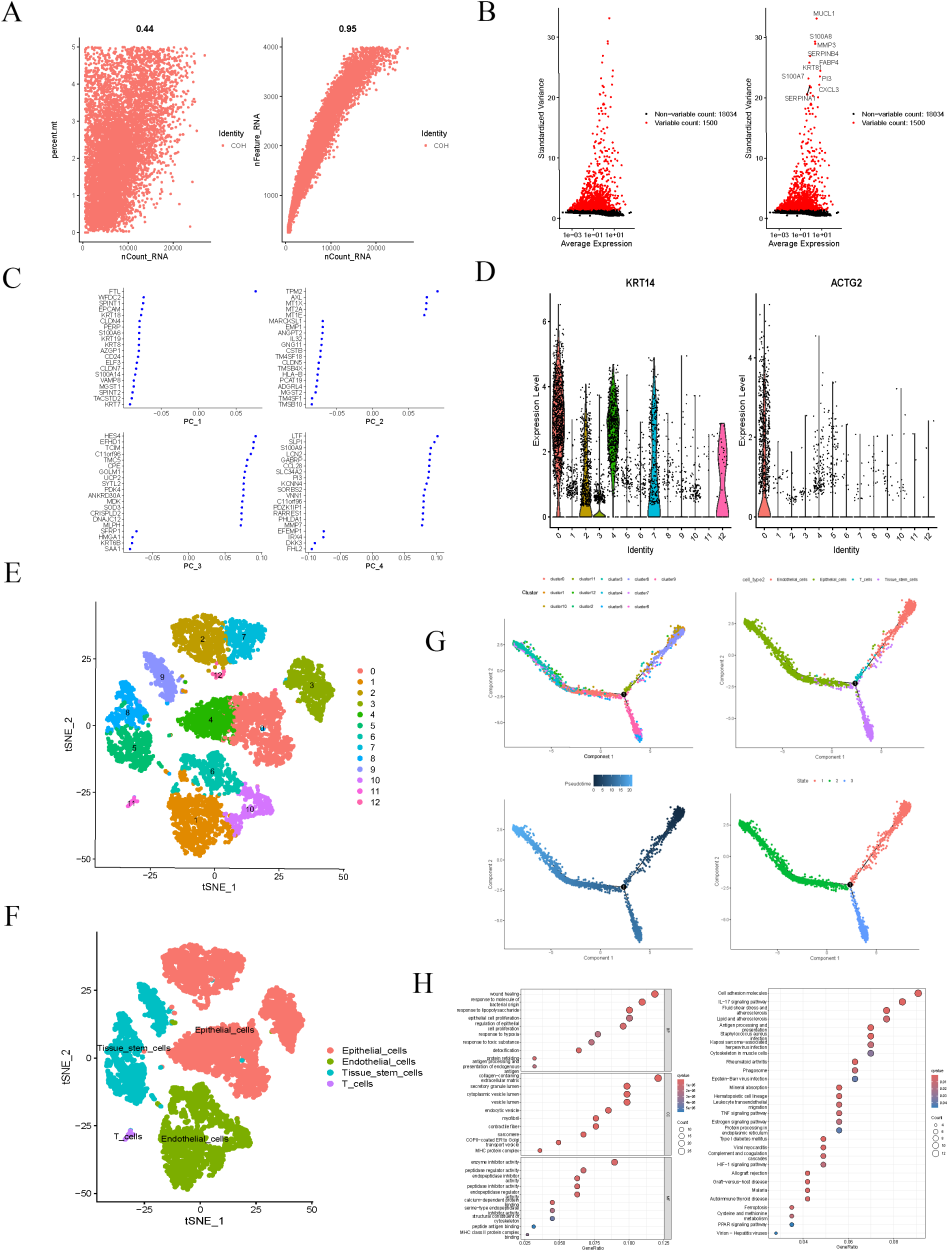
Single-cell analysis. **(A)** These two scatter plots were used to assess the quality of the single-cell RNA sequencing data. **(B)** The top 1500 genes with the highest expression variability were selected, and the top 10 genes were ranked and highlighted. **(C)** The characteristic genes of PC1-PC4 are displayed. **(D)** The violin plot for marker genes. **(E)** The UMAP plot shows all cells divided into 12 clusters. **(F)** Breast cancer samples are classified into four distinct cell types. **(G)** The cell differentiation trajectory plot. **(H)** GO pathways involve biological processes in BP, MF, and CC, as well as significant enriched pathways in KEGG.

We performed stringent quality control on the single-cell RNA sequencing data and assessed data quality through scatter plots to ensure the reliability of subsequent analyses (**Figure 1A**). We selected the top 1, 500 most variable genes based on gene expression variability, highlighting the top 10 of these highly variable genes (**Figure 1B**), which were deemed significant biological relevance for further analysis. Using PCA, we extracted the first four principal components (PC1-PC4) and displayed the characteristic genes for each element (**Figure 1C**). These characteristic genes provided crucial information for cell clustering and classification. Next, we utilized violin plots to illustrate the expression distribution of marker genes within the cells, further validating the specificity of these genes in cell type identification (**Figure 1D**). Employing the UMAP dimensionality reduction algorithm, we classified all cells into 12 clusters (**Figure 1E**). Further analysis indicated that these cells could be categorized into four main cell types: epithelial cells, endothelial cells, tissue stem cells, and T cells (**Figure 1F**). This classification result revealed the high heterogeneity of cells within BC tissue, providing a clear cellular framework for subsequent studies. Cell trajectory analysis delved into the differentiation pathways of these cells (**Figure 1G**), revealing significant differences in gene expression among various cell types during differentiation. These differential genes may play crucial roles at different differentiation stages, thereby providing important insights for further investigations into the differentiation mechanisms of BC cells. We conducted GO and KEGG pathway enrichment analyses for the differential genes identified during cell differentiation. GO analysis indicated that these genes were significantly enriched in key biological processes such as healing, antigen activity, and enzyme inhibition (**Figure 1H**). KEGG analysis further revealed the significant roles of these genes in cancer-related pathways, such as cell adhesion molecules, the IL-17 signaling pathway, and fluid shear stress (**Figure 1H**). These enrichment analysis results provide a vital foundation for understanding the molecular mechanisms underlying the differentiation of BC cells.

### 3.2 Molecular subtypes of BC and immune microenvironment analysis

**Figure 2:**
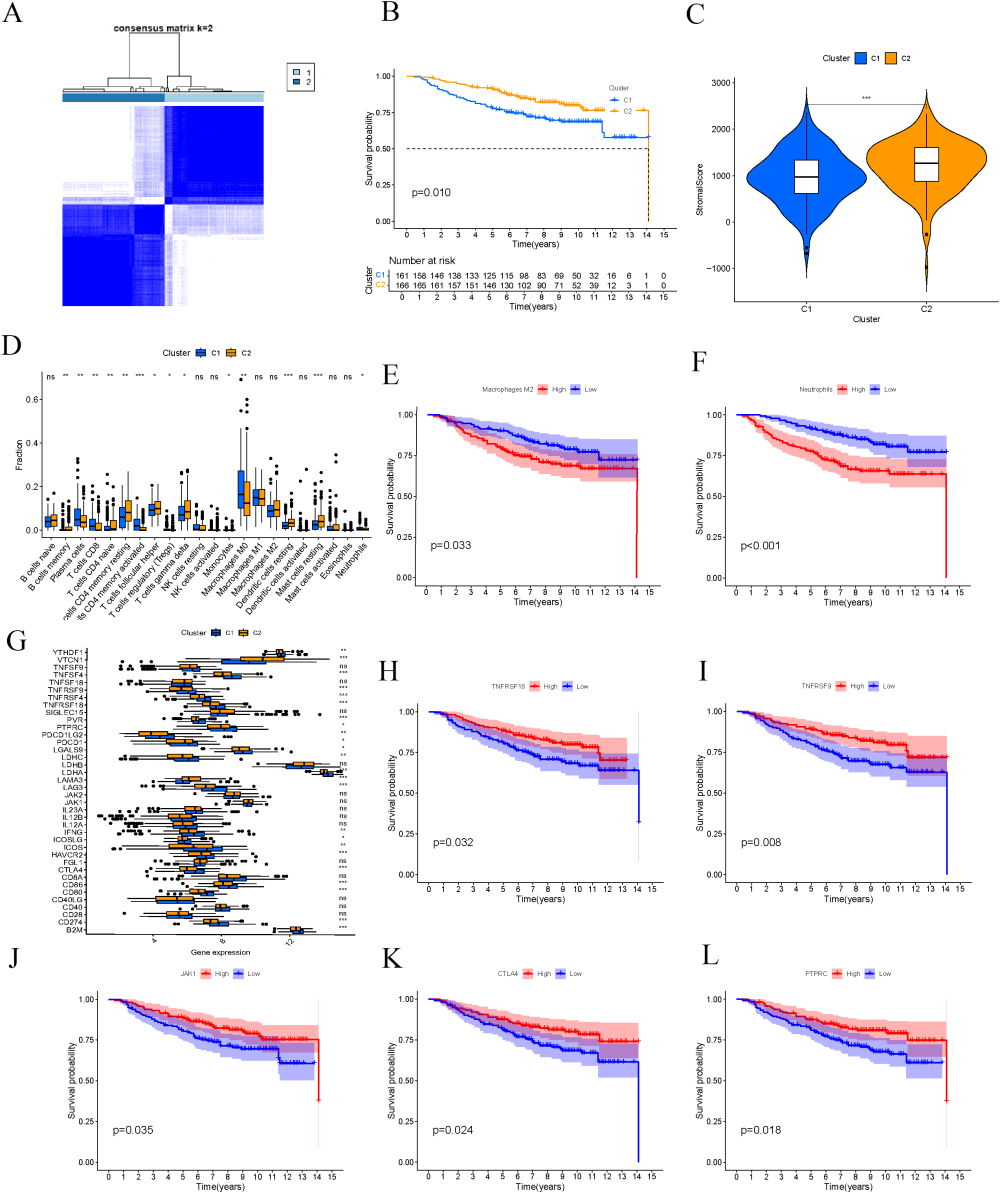
Molecular subtypes of breast cancer. **(A)** Breast cancer patients were divided into two subgroups through consensus clustering analysis. **(B)** Survival curves of the two clusters are depicted. **(C)** Differential analysis of immune cells in different clusters. **(D)** Differential analysis of immune checkpoint genes in different clusters. **(E, F)** Comparison of survival differences between high- and low-infiltration breast cancer patients. **(H–L)** Survival curves of breast cancer patients based on the expression levels of specific immune checkpoint genes. ns, not significant; *, p < 0.05; **, p < 0.01; ***, p < 0.001.

Through consensus clustering analysis, we classified BC patients into two molecular subtypes (**Figure 2A**). Survival curve analysis demonstrated a significant difference in survival rates between these two subtypes, with patients in the C1 group exhibiting poorer prognoses compared to those in the C2 group (**Figure 2B**), suggesting that molecular subtypes may serve as important prognostic indicators for BC patients. We assessed the stromal scores of both subtypes, revealing that the C2 group had a higher stromal score, and the level of stromal cell infiltration within the tumor tissue was also significantly elevated (**Figure 2C**). Further analysis of immune cell infiltration differences between subtypes indicated a higher level of immune-activating cells (e.g., T cells) in the C2 group. In contrast, the C1 group showed more pronounced infiltration of immune-suppressive cells (e.g., regulatory T cells and neutrophils) (**Figure 2D**). By examining the survival outcomes of BC patients with varying levels of immune cell infiltration, we discovered that higher levels of neutrophils and M2 macrophages were significantly linked to worse prognoses (**Figures 2E, F**). This finding further emphasizes the critical role of the immune microenvironment in BC prognosis. Additionally, we assessed the expression levels of immune checkpoint genes across the two subtypes, revealing significant differences for specific genes. These discrepancies suggest that these immune checkpoint genes may be crucial for BC immunotherapy (**Figure 2G**). We also plotted survival curves based on the expression levels of specific immune checkpoint genes, including TNFRSF18, TNFRSF9, CTLA4, PTPRC, and JAK1. The findings showed that patients with high expression of these immune checkpoint genes generally had better survival outcomes (**Figures 2H–L**), indicating that these genes might be associated with improved responses and efficacy in immunotherapy.

### 3.3 Development and evaluation of a prognostic model for BC utilizing centromere-associated genes

**Figure 3:**
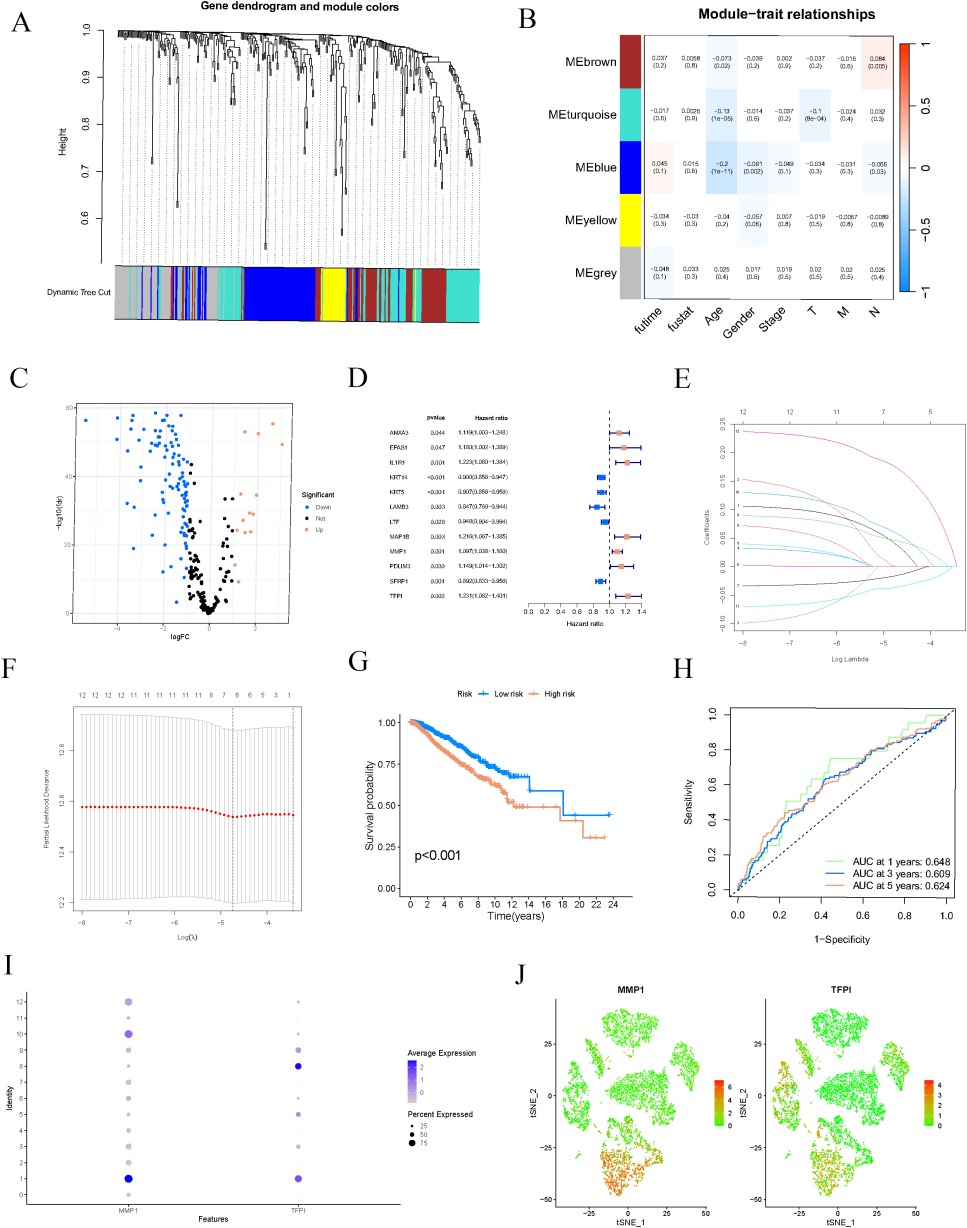
Construction of the prognostic model. **(A)** The gene dendrogram illustrates the clustering of genes based on their expression patterns, with different colors representing different gene modules. **(B)** The module-trait relationship heatmap displays the correlation and significance between different gene modules and clinical features (such as tumor stage, age, sex, etc.). **(C)** Volcano plot of differentially expressed genes between breast cancer and normal samples. **(D)** Univariate Cox regression forest plot. **(E, F)** LASSO-Cox regression analysis of potential prognostic genes in the training cohort to develop the prognostic risk features. **(G)** The K-M curve for the training group shows the prognosis of breast cancer patients in high-risk and low-risk groups. **(H)** ROC curves for the one-year, three-year, and five-year cohorts. **(I, J)** Expression of model genes in the 12 clusters.

Using WGCNA, we developed a gene co-expression network and conducted module clustering according to the expression patterns observed (**Figure 3A**). The module-trait relationship heatmap indicated that specific gene modules were significantly associated with clinical features of BC, such as tumor stage, age, and gender (**Figure 3B**), suggesting that these modules could play essential roles in the occurrence and progression of BC. We compared gene expression differences between BC and standard samples, depicting the significant differential genes using a volcano plot (**Figure 3C**). Subsequently, we performed an intersection analysis of the three selected WGCNA modules (blue, brown, and turquoise) with centromere-related genes and the differential genes identified in the previous cell trajectory analysis, thereby narrowing down the range of key genes. We conducted univariate Cox regression analysis to pinpoint genes significantly linked to BC prognosis (**Figure 3D**). Next, we utilized LASSO-Cox regression analysis to identify MMP1 and TFPI as critical indicators for constructing the prognostic model from the candidate genes (**Figures 3E, F**). We categorized BC patients into high-risk and low-risk categories. Kaplan-Meier survival curves revealed that the high-risk patients experienced significantly worse outcomes than their low-risk counterparts (**Figure 3G**), confirming the model’s validity. Additionally, ROC curve analysis showed that the model accurately predicted survival rates at 1, 3, and 5 years, with AUC values of 0.648, 0.609, and 0.624, respectively (**Figure 3H**). To explore the roles of MMP1 and TFPI in the tumor microenvironment, we analyzed their expression distributions at the single-cell level. The results demonstrated that MMP1 was highly expressed in cell cluster 1. In contrast, TFPI was significantly expressed in cell cluster 8 (**Figure 3I-J**), suggesting their potential involvement in BC progression and immune regulation through different mechanisms.

### 3.4 Assessment of the model’s independent prognostic significance and the development of a nomogram were performed

**Figure 4:**
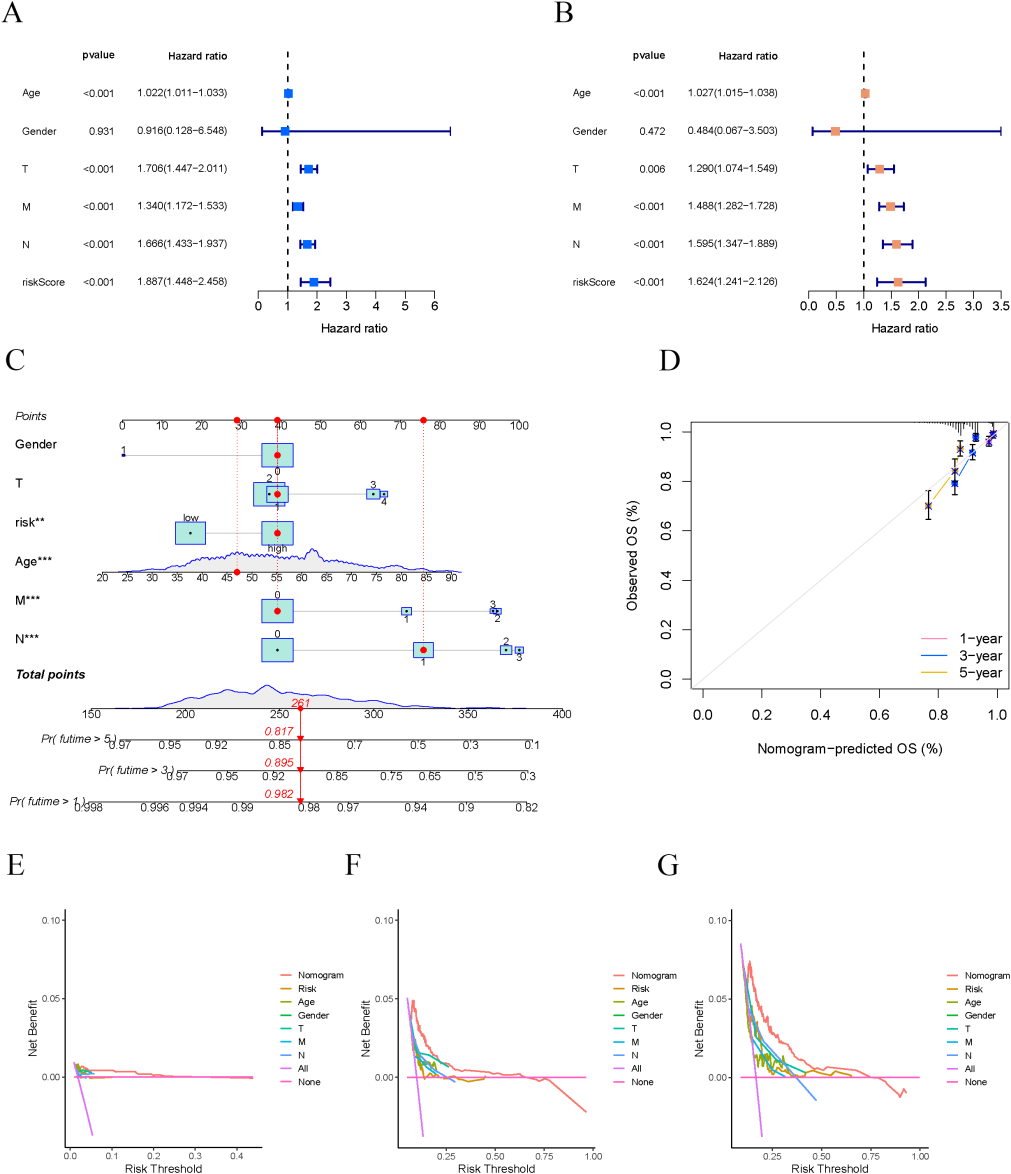
Construction and validation of the prognostic nomogram. **(A, B)** Univariate and multivariate Cox regression forest plots for risk scores and clinical characteristics. **(C)** Nomogram validation for overall survival in breast cancer patients. **(D)** Calibration curve to validate the predictive ability of the nomogram. **(E-G)** DCA curves for risk scores and clinical features (1-year, 3-year, and 5-year).

The univariate Cox regression analysis revealed a significant relationship between the risk score and the overall survival of BC patients (**Figure 4A**). Multivariate Cox regression analysis further supported this result, demonstrating that the risk score remained an independent prognostic factor for BC patients even after adjusting for clinical characteristics such as age, tumor stage, and gender (**Figure 4B**). These findings underscore the clinical significance of the prognostic model based on centromere-related genes in predicting survival outcomes for BC patients. To enhance the application of this model in clinical practice, we integrated the risk score with other key clinical features (e.g., age and tumor stage) to construct a nomogram for predicting overall survival in BC patients (**Figure 4C**). This nomogram utilizes an intuitive scoring system to assist clinicians in rapidly assessing the prognostic risk of patients. The calibration curve analysis confirmed the nomogram’s reliability in forecasting survival rates at 1, 3, and 5 years (**Figure 4D**), demonstrating a strong alignment between predicted and observed outcomes. This suggests that the nomogram delivers robust predictive capabilities. Moreover, the findings indicated that this nomogram, which incorporates risk scores and clinical characteristics, surpassed traditional clinical models in estimating patient survival outcomes over the 1-year, 3-year, and 5-year marks (**Figures 4E–G**). This implies that the nomogram offers more dependable guidance for clinical decisions, enhancing treatment strategies for patients.

### 3.5 Analysis of functional enrichment was conducted on the differentially expressed genes between the high-risk and low-risk groups

**Figure 5:**
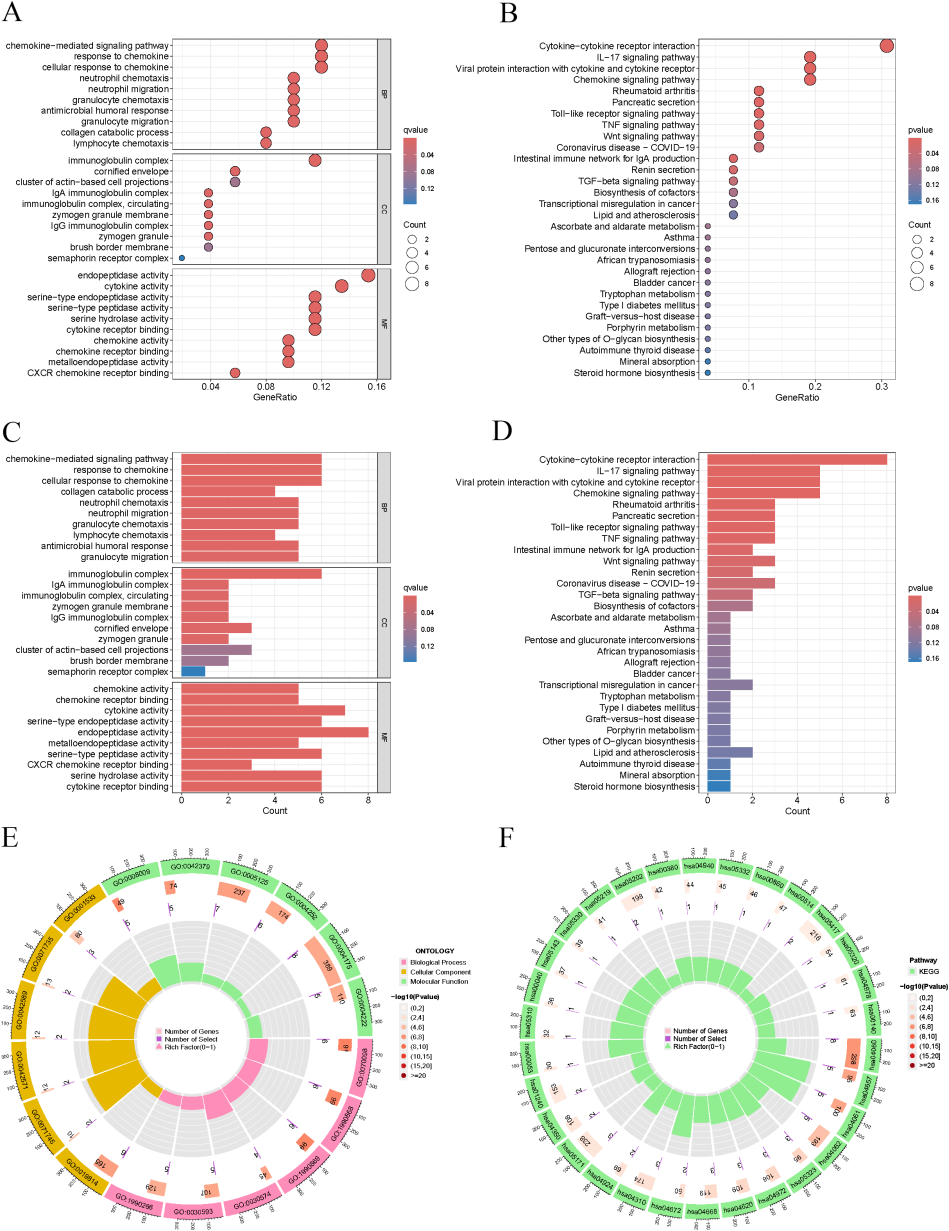
Enrichment analysis. **(A, C, E)** GO pathways involving biological processes in BP, MF, and CC. **(B, D, F)** Significantly enriched pathways in KEGG.

The results of the GO analysis indicated that the differentially expressed genes were significantly enriched in critical biological processes, including cytokine-mediated signaling pathways, immunoglobulin complexes, and endopeptidase activity (**Figures 5A, C, E**). The KEGG analysis provided insights into the essential functions of these differentially expressed genes within various tumor-related signaling pathways. Notably, enriched pathways included interactions between cytokines and their receptors, IL-17 signaling, viral protein interactions with cytokines and their receptors, and chemokine signaling pathways (**Figures 5B, D, F**).

### 3.6 Immune characteristics analysis of high-risk and low-risk patients

**Figure 6:**
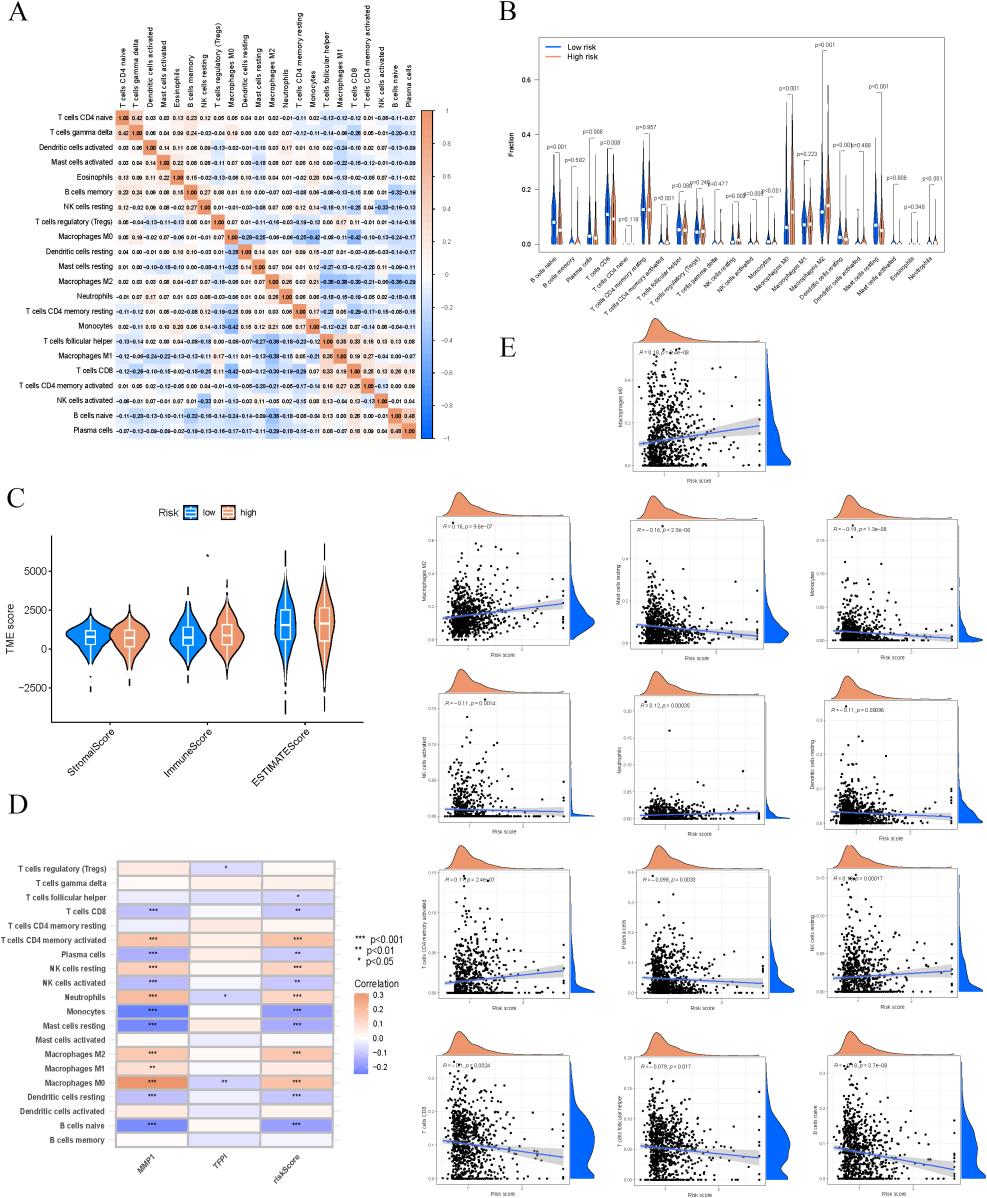
Different immune characteristics in risk groups. **(A)** Correlation heatmap of immune cells. **(B)** Differential analysis of immune cells in different risk groups. **(C)** Differential assessment of tumor microenvironment scores. **(D)** Correlation strength between model genes and immune cell types. **(E)** Significant correlation between immune cells and risk scores. ns, not significant; *, p < 0.05; **, p < 0.01; ***, p < 0.001.

Utilizing a heatmap to illustrate immune cell correlations, we examined the expression patterns of various immune cell types in the high-risk and low-risk groups (**Figure 6A**). We also compared immune cell infiltration levels between these two groups (**Figure 6B**). The analysis showed that the high-risk group had significantly higher levels of infiltration for M0 macrophages, M2 macrophages, and NK cells. In contrast, the low-risk group exhibited markedly increased infiltration of activated CD8 T cells and resting mast cells. These results highlighted substantial differences in immune cell composition across the risk groups. Additionally, we assessed the tumor microenvironment scores for both groups (**Figure 6C**). The findings revealed that the high-risk group had a significantly elevated immune score compared to the low-risk group. This suggests that the tumor microenvironment in the high-risk group is characterised by increased immune cell infiltration, which may be connected to worse prognostic outcomes. Additionally, we analysed the correlation of two key genes (MMP1 and TFPI) with immune cell types in the model (**Figure 6D**). The findings indicated a notable positive correlation between MMP1 and M0 macrophages as well as neutrophils. Conversely, TFPI showed a significant negative correlation with both M0 macrophages and neutrophils. These results suggest that MMP1 and TFPI may influence the characteristics of the tumor microenvironment by regulating immune cell infiltration. Correlation analyses indicated that the risk score was significantly associated with various immune cell types (**Figure 6E**). For instance, the risk score was positively correlated with M0 macrophages and activated CD4 memory cells, whereas it displayed a negative correlation with monocytes and resting mast cells. These results further highlight the significant role of immune cells in the prognosis of BC.

### 3.7 Chemosensitivity analysis of high-risk and low-risk BC patients

**Figure 7:**
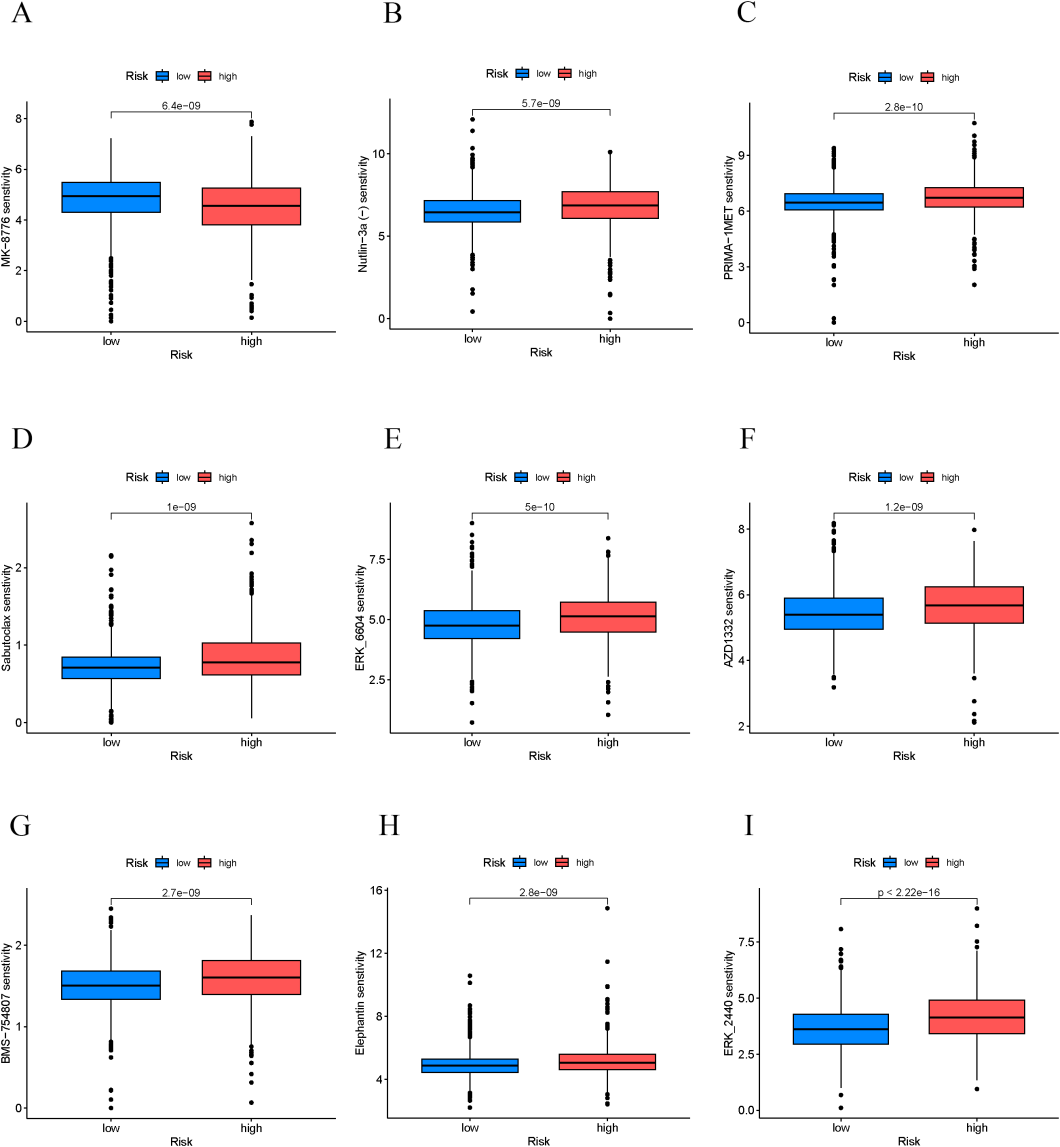
Prediction of chemotherapy drug sensitivity in breast cancer patients. **(A–I)** IC50 values of chemotherapy drugs in high/low-risk group patients.

We assessed the sensitivity differences of high-risk and low-risk patients to various chemotherapy drugs by calculating the IC50 values (**Figures 7A–I**). The results indicated that high-risk patients had significantly higher IC50 values for certain chemotherapy drugs (e.g., ERK_2440, ERK_6604, and PRIMA−1MET) compared to low-risk patients, suggesting that high-risk patients demonstrated lower sensitivity to these drugs and potentially higher levels of resistance. Conversely, high-risk patients showed significantly lower IC50 values for some drugs (e.g., MK−8776), which implies that these medications may hold greater therapeutic potential for high-risk patients. This finding offers new options for personalized treatment strategies for the high-risk group. Our analysis suggests that high-risk patients exhibited lower sensitivity to traditional chemotherapy drugs, which may be associated with their poorer prognosis. However, potential effective drugs identified for the high-risk group (e.g., ERK_2440) may provide more effective treatment alternatives. These results have significant implications for the selection of clinical treatment regimens.

### 3.8 Molecular docking and screening of potential therapeutic compounds

**Figure 8:**
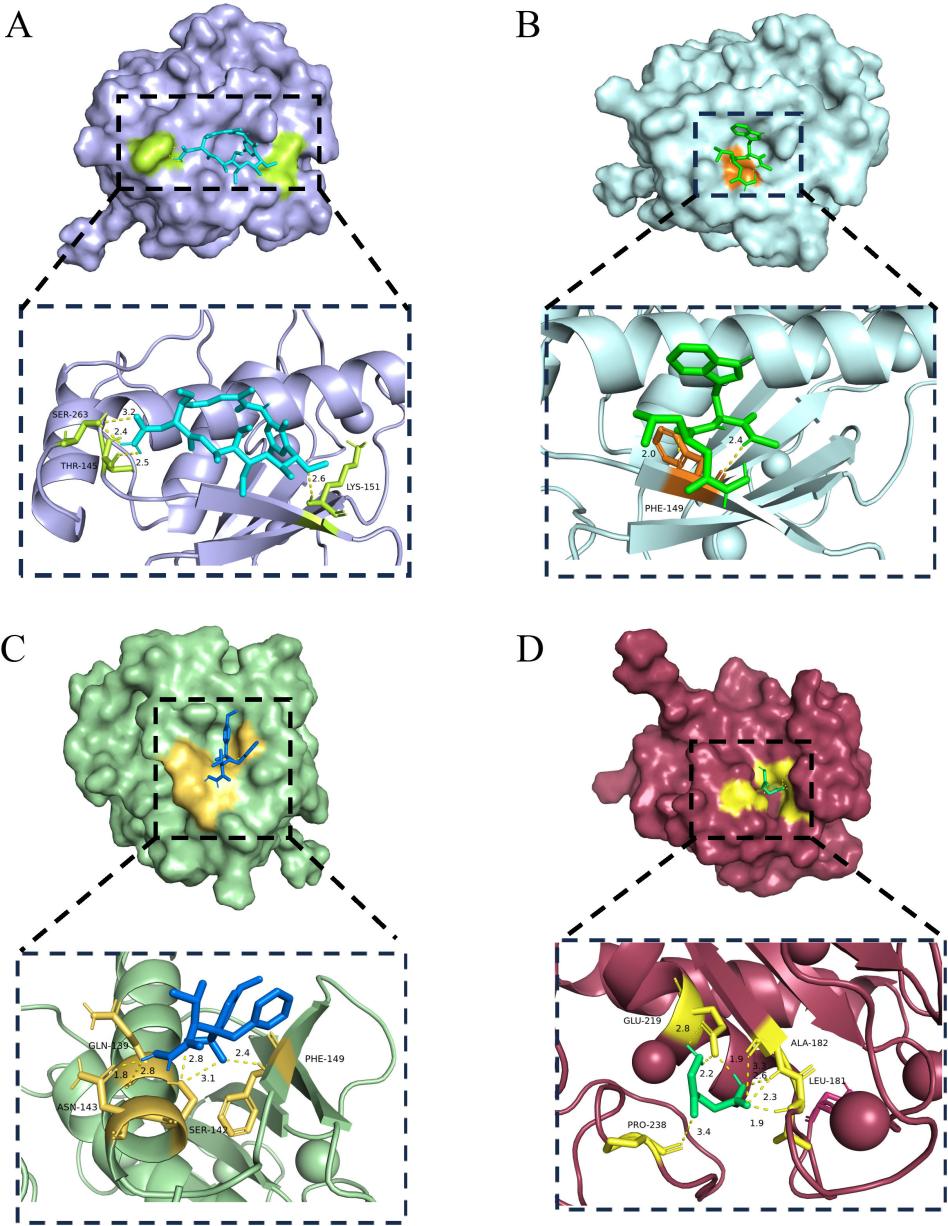
Potential therapeutic compounds for MMP1 and molecular docking analysis.​​ **(A-D)**​​ Molecular docking was performed for the top four candidate drugs (herbimycin A, ilomastat, CGS-27023A, and aminolevulinic acid).

In our prognostic model, the MMP1 gene emerged as a significant biomarker influencing the overall survival of BC patients. To investigate potential therapeutic strategies targeting this gene, we performed extensive drug screenings utilizing various drug prediction platforms. By integrating adjusted p-values, we identified multiple compounds with possible therapeutic benefits. We then evaluated the binding affinity of these selected drugs through molecular docking studies focused on the top four candidates. Particular attention was given to the candidate with the lowest adjusted p-value and its interaction sites with the MMP1 protein. By calculating the binding energies, we generated a series of docking results (**Figures 8A–D**). Among these results, the binding energy between MMP1 and herbimycin A was the lowest at -6.6 kcal/mol, indicating a very stable interaction. This finding not only aids in predicting potentially effective drugs but also deepens our understanding of the interactions between the drug and its target, providing valuable information for drug development and optimization.

### 3.9 Expression and prognostic value of MMP1 in breast tissue

**Figure 9:**
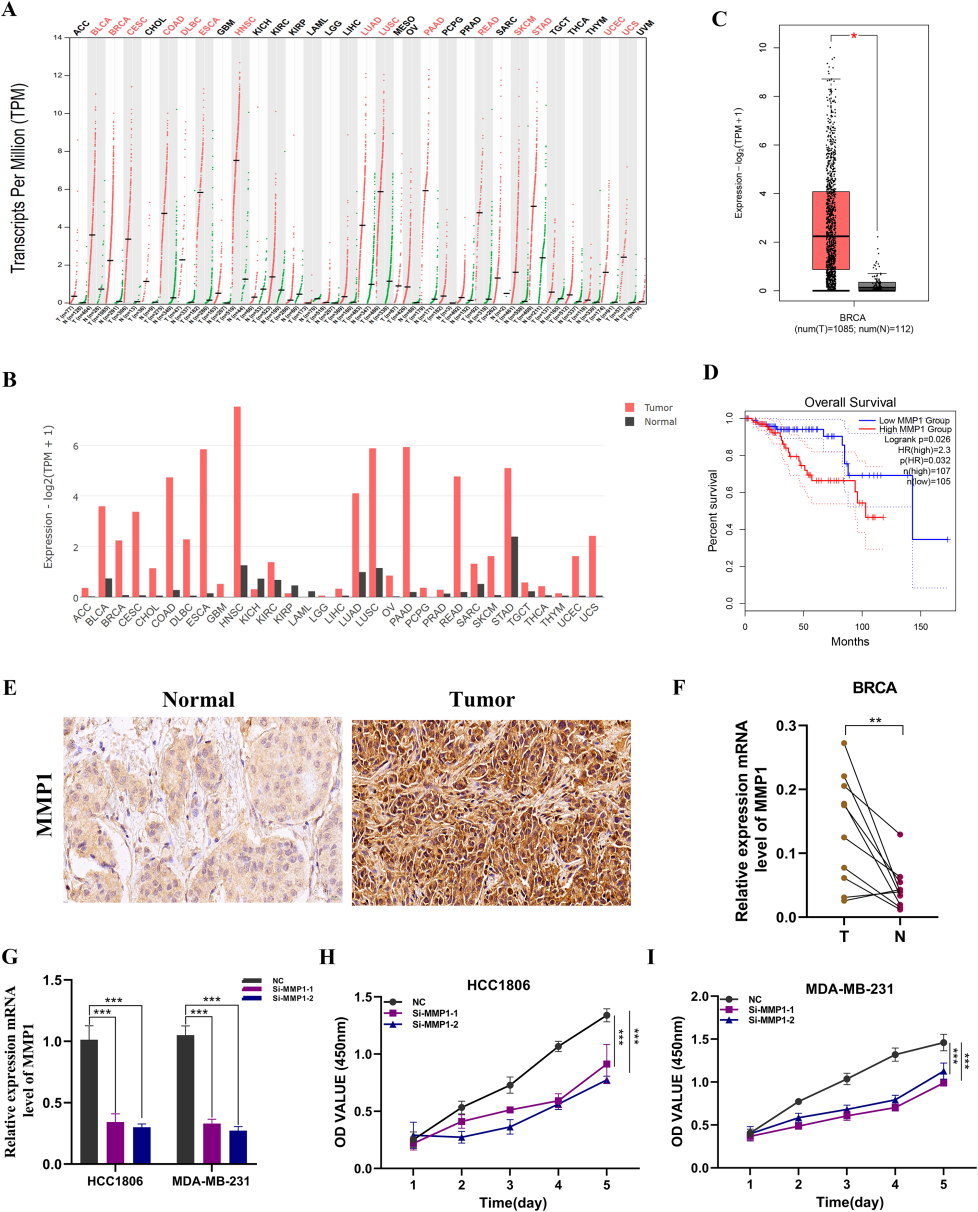
Expression analysis and experimental validation of MMP1. **(A, B)** Pan-cancer expression profile of MMP1. **(C)** Expression level of MMP1 in breast cancer samples. **(D)** Overall survival (OS) analysis of MMP1 in the TCGA breast cancer cohort. **(E)** Immunohistochemical analysis of MMP1 in breast cancer and adjacent normal tissues. **(F)** PCR validation in clinical samples showing high expression of MMP1 in breast cancer (BC). **(G)** Knockdown efficiency of MMP1 in HCC1806 and MDA-MB-231 cell lines. **(H, I)** CCK-8 assay demonstrating that MMP1 knockdown significantly reduces cell proliferation. ns, not significant; *, p < 0.05; **, p < 0.01; ***, p < 0.001.

Pan-cancer analysis of MMP1 indicated that it is highly expressed in most tumors compared to normal tissues (**Figures 9A, B**). In BC samples, MMP1 expression levels were also elevated (**Figure 9C**). To further verify the prognostic effect of MMP1, we conducted overall survival (OS) analysis, revealing that patients with high MMP1 expression had poorer prognoses (P < 0.05, **Figure 9D**). Subsequently, we performed immunohistochemical validation on BC and adjacent normal tissue samples, which showed significantly higher levels of MMP1 expression in BC tissues (**Figure 9E**). Additionally, analysis of MMP1 mRNA levels in ten pairs of BC tissue samples confirmed a similar expression trend (**Figure 9F**), thus validating the accuracy of the bioinformatics study.

### 3.10 Functional validation of MMP1

**Figure 10:**
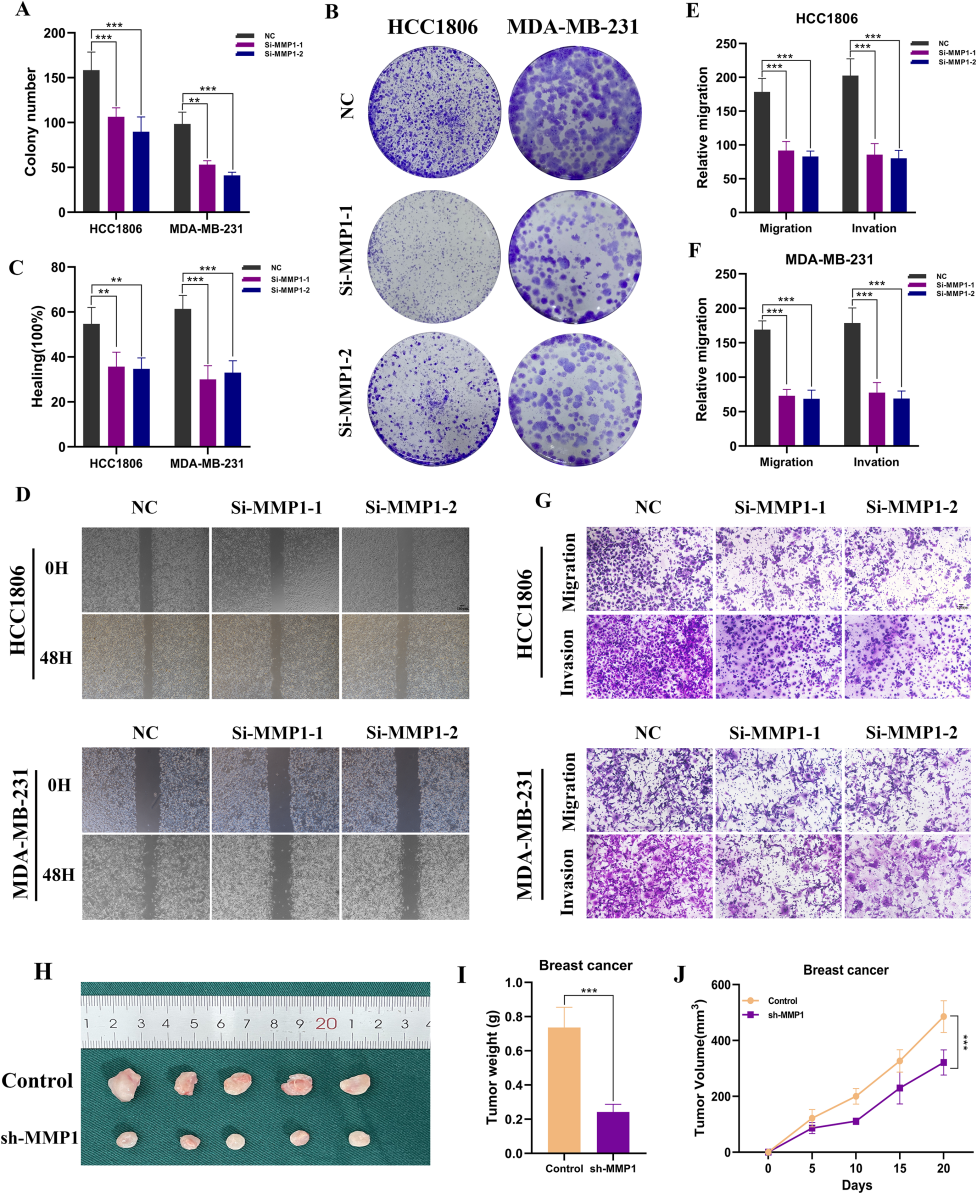
Functional assays following MMP1 knockdown. **(A, B)** Colony formation assays showing significantly reduced clonogenic ability of MDA-MB-231 and HCC1806 cells after MMP1 knockdown. **(C, D)** Wound healing assays demonstrating impaired migratory capacity in both cell lines following MMP1 silencing. **(F, G)** Transwell assays indicating markedly decreased migration and invasion abilities in MDA-MB-231 and HCC1806 cells after MMP1 knockdown. **(H)** Representative images of subcutaneous tumors excised from nude mice injected with sh-NC or sh-MMP1–transfected cells. **(I, J)** Average tumor weight and volume comparison between groups.

We conducted MMP1 knockdown experiments in MDA-MB-231 and HCC1806 cell lines, resulting in significant changes in MMP1 expression levels in both cell lines (**Figure 9G**). In CCK-8 assays, we observed a notable decrease in the proliferation activity of MDA-MB-231 and HCC1806 cells following MMP1 knockout compared to control cells (**Figures 9H, I**). To further validate the impact of MMP1 on BC cell proliferation, we performed colony formation assays, which indicated that MMP1 gene knockdown led to reduced colony number and size in both cell lines (**Figures 10A, B**). We then examined the impact of MMP1 on the migration and invasion of BC cells through wound healing and transwell assays. The findings revealed that the knockdown of MMP1 significantly reduced the migration and invasion capacities of these cancer cells (**Figures 10C–G**). *In vivo* experiments also showed that MMP1 knockdown inhibited tumor growth in mice compared to the control group, leading to reductions in tumor volume and weight (**Figures 10H–J**).

## 4 Discussion

Even with significant progress in the early detection of BC, surgical methods, and various systemic treatments—including hormone therapy, chemotherapy, targeted therapies, and immunotherapy—challenges like recurrence, metastasis, and resistance to treatment persist (1, 33). The core obstacle to effective therapy lies in the intrinsic heterogeneity of BC. This heterogeneity spans multiple levels, including molecular subtypes (e.g., Luminal, HER2-positive, and triple-negative breast cancer), genomic alterations (such as PIK3CA mutations and BRCA1/2 deletions), and features of the tumor microenvironment (e.g., immune cell infiltration and stromal fibrosis). Collectively, these variations contribute to markedly different treatment responses, resulting in highly individualized clinical outcomes and prognoses (34–37). However, their efficacy remains limited by the absence of robust predictive biomarkers and the complexity of resistance mechanisms. In this context, early and accurate diagnosis, along with tailored therapeutic strategies, is critical for improving patient outcomes. Yet, current diagnostic and prognostic approaches still rely heavily on clinical manifestations and histopathological evaluation, which fall short in capturing the underlying tumor heterogeneity and evolutionary dynamics. Traditional clinical markers (such as ER/PR, HER2, and Ki-67) primarily reflect tumor characteristics such as hormone receptor status and proliferation rate, but they may not fully capture the complexity of BC, especially in more aggressive subtypes like triple-negative breast cancer. In contrast, our CENP-based model integrates genomic instability and chromosomal segregation defects, providing a more comprehensive view of tumor biology (23, 38, 39).

This study investigates centromere proteins (CENPs), crucial for chromosome segregation, and their prognostic significance in BC. We developed a prognostic model using CENP-associated genes by integrating transcriptomic and single-cell RNA sequencing data from TCGA and GEO. Key indicators identified through bioinformatics methods include MMP1 and TFPI, achieving AUC values of 0.648, 0.609, and 0.624 in distinguishing high- from low-risk patients. Kaplan-Meier analysis indicated that high-risk groups had significantly poorer survival, supported by multivariate Cox regression identifying the risk score as an independent prognostic marker. Furthermore, we analyzed the tumor immune microenvironment and found that high-risk tumors had lower immune cell infiltration and higher tumor purity, suggesting enhanced tumor evasion from immune surveillance. These insights may aid in risk stratification and personalized treatment for BC.

Furthermore, we noted variations in the expression of various immune checkpoint-related genes between the high-risk and low-risk groups. The overall trend suggests a strong association between the risk score and the immunological status of the tumor. This finding holds important clinical implications, as the tumor immune landscape is a critical determinant of immunotherapy efficacy. It provides a rationale for tailoring individualized immunotherapeutic strategies according to patient risk stratification. Specifically, patients with a low-risk profile may benefit more from immune checkpoint blockade, while alternative or combination approaches may be needed for high-risk patients (40–45). Our drug sensitivity analysis further revealed that high-risk patients exhibited reduced responsiveness to several commonly used chemotherapeutic agents, such as anthracyclines and taxanes. This suggests that tumors in the high-risk group may have a higher degree of chemoresistance. Therefore, traditional chemotherapy regimens alone may be insufficient for these patients, and novel targeted therapies or combinatorial treatment strategies may be more beneficial. These findings offer valuable guidance for clinical decision-making and support the development of risk-adapted, personalized treatment plans for BC patients.

MMP1, one of the core genes included in our prognostic model, belongs to the matrix metalloproteinase (MMP) family and primarily functions in the degradation of extracellular matrix (ECM) components (46, 47). In BC, MMP1 is frequently overexpressed and is significantly correlated with enhanced tumor invasiveness and metastatic potential. High MMP1 expression not only predicts poorer clinical outcomes but may also facilitate disease progression by remodeling the tumor microenvironment (48, 49). Previous studies have reported a strong association between MMP1 expression levels and the infiltration of immune effector cells, including CD8^+^ T cells, macrophages, and dendritic cells. This suggests that MMP1-mediated ECM degradation and associated signaling events may influence immune cell recruitment and distribution within the tumor, thereby modulating the immune landscape (50, 51). Additionally, upregulation of MMP1 has been implicated in the acquisition of chemoresistance. Specifically, high MMP1 levels in BC have been shown to reduce the sensitivity of tumor cells to chemotherapeutic agents, whereas MMP1 inhibition significantly restores drug responsiveness in resistant cancer cells (52). Consistent with these findings, our study confirmed that MMP1 is markedly overexpressed in BC tissues. Functional experiments further demonstrated that knockdown of MMP1 significantly suppressed the viability, invasiveness, and migratory capacity of BC cells. *In vivo* experiments in mice also revealed that silencing MMP1 effectively inhibited tumor growth. These findings highlight the crucial involvement of MMP1 in the progression of BC and suggest that it may be a promising target for therapeutic intervention.

While this study yielded significant findings, it has limitations that future research should address. The prognostic model’s development relied on retrospective data from public databases without validation in prospective clinical cohorts, raising concerns about generalizability due to batch effects and patient heterogeneity. Thus, further validation in multi-center BC cohorts is essential for confirming the model’s robustness. Additionally, the model is based on only two genes, which simplifies its application but may limit the biological insights. Future studies should consider incorporating more prognostically relevant genes or multi-omics data—such as genomic, epigenetic, and proteomic profiles—to better capture tumor heterogeneity and enhance predictive power. Moreover, future studies should aim to validate this prognostic model in multi-center prospective cohorts to confirm its robustness and applicability in clinical practice.

## 5 Conclusions

In this study, we investigated the expression patterns and prognostic significance of centromere-associated genes in BC using multi-cohort datasets from TCGA and GEO, along with single-cell RNA sequencing and other analytical techniques. We developed a CENP-related prognostic model consisting of two genes, MMP1 and TFPI, which demonstrated strong potential for stratifying BC patients by prognosis and molecular subtype. This model provides a foundation for personalized treatment strategies, especially for high-risk populations, and shows promise for future clinical application.

## Data Availability

The datasets presented in this study can be found in online repositories. The names of the repository/repositories and accession number(s) can be found in the article/**Supplementary Material**.

## References

[1] Xiong X Zheng LW Ding Y Chen YF Cai YW Wang LP . Breast cancer: pathogenesis and treatments. Signal Transduct Target Ther. (2025) 10:49. doi: 10.1038/s41392-024-02108-4, PMID: 39966355 PMC11836418

[2] HendersonJT WebberEM WeyrichMS MillerM MelnikowJ . Screening for breast cancer: evidence report and systematic review for the US preventive services task force. JAMA. (2024) 331:1931–46. doi: 10.1001/jama.2023.25844, PMID: 38687490

[3] Siegel RL Kratzer TB Giaquinto AN Sung H Jemal A . Cancer statistics, 2025. CA Cancer J Clin. (2025) 75:10–45. doi: 10.3322/caac.21871, PMID: 39817679 PMC11745215

[4] Lin CJ Jin X Ma D Chen C Ou-Yang Y Pei YC . Genetic interactions reveal distinct biological and therapeutic implications in breast cancer. Cancer Cell. (2024) 42:701–719.e12. doi: 10.1016/j.ccell.2024.03.006, PMID: 38593782

[5] Montazeri Aliabadi H . Molecular targets for breast cancer therapy. Biomolecules. (2024) 14:10. doi: 10.3390/biom14101219, PMID: 39456152 PMC11506731

[6] Qin W Li J Gao N Kong X Guo L Chen Y . Multiomics-based molecular subtyping based on the commensal microbiome predicts molecular characteristics and the therapeutic response in breast cancer. Mol Cancer. (2024) 23:99. doi: 10.1186/s12943-024-02017-8, PMID: 38730464 PMC11083817

[7] Jiang B Bao L He S Chen X Jin Z Ye Y . Deep learning applications in breast cancer histopathological imaging: diagnosis, treatment, and prognosis. Breast Cancer Res. (2024) 26:137. doi: 10.1186/s13058-024-01895-6, PMID: 39304962 PMC11416021

[8] Yan Q Deng Y Zhang Q . A comprehensive overview of metaplastic breast cancer: Features and treatments. Cancer Sci. (2024) 115:2506–14. doi: 10.1111/cas.16208, PMID: 38735837 PMC11309924

[9] Zhang Y Chen F Balic M Creighton CJ . An essential gene signature of breast cancer metastasis reveals targetable pathways. Breast Cancer Res. (2024) 26:98. doi: 10.1186/s13058-024-01855-0, PMID: 38867323 PMC11167932

[10] Shan R Dai LJ Shao ZM Jiang YZ . Evolving molecular subtyping of breast cancer advances precision treatment. Cancer Biol Med. (2024) 21:731–9. doi: 10.20892/j.issn.2095-3941.2024.0222, PMID: 39302031 PMC11414217

[11] Xiao C Guo Y Xu Y Huang J Li J . Clinicopathological characteristics and survival analysis of different molecular subtypes of breast invasive ductal carcinoma achieving pathological complete response through neoadjuvant chemotherapy. World J Surg Oncol. (2024) 22:250. doi: 10.1186/s12957-024-03535-x, PMID: 39285422 PMC11403885

[12] Rayson VC Harris MA Savas P Hun ML Virassamy B Salgado R . The anti-cancer immune response in breast cancer: current and emerging biomarkers and treatments. Trends Cancer. (2024) 10:490–506. doi: 10.1016/j.trecan.2024.02.008, PMID: 38521654

[13] Chen W Kang Y Sheng W Huang Q Cheng J Pei S . A new 4-gene-based prognostic model accurately predicts breast cancer prognosis and immunotherapy response by integrating WGCNA and bioinformatics analysis. Front Immunol. (2024) 15:1331841. doi: 10.3389/fimmu.2024.1331841, PMID: 38370403 PMC10869553

[14] Mei W Faraj Tabrizi S Godina C Lovisa AF Isaksson K Jernstrom H . A commonly inherited human PCSK9 germline variant drives breast cancer metastasis via LRP1 receptor. Cell. (2025) 188:371–389.e28. doi: 10.1016/j.cell.2024.11.009, PMID: 39657676 PMC11770377

[15] Mahlke MA Nechemia-Arbely Y . Guarding the genome: CENP-A-chromatin in health and cancer. Genes (Basel). (2020) 11:7. doi: 10.3390/genes11070810, PMID: 32708729 PMC7397030

[16] Renaud-Pageot C Quivy JP Lochhead M Almouzni G . CENP-A regulation and cancer. Front Cell Dev Biol. (2022) 10:907120. doi: 10.3389/fcell.2022.907120, PMID: 35721491 PMC9201071

[17] Pan T Zhou D Shi Z Qiu Y Zhou G Liu J . Centromere protein U (CENPU) enhances angiogenesis in triple-negative breast cancer by inhibiting ubiquitin-proteasomal degradation of COX-2. Cancer Lett. (2020) 482:102–11. doi: 10.1016/j.canlet.2019.11.003, PMID: 31705927

[18] Chen H Pu S Yu S Liao X He J Zhang H . A nomogram based on CENPP expression for survival prediction in breast cancer. Gland Surg (2021) 10:1874–88. doi: 10.21037/gs-21-30, PMID: 34268072 PMC8258876

[19] Liu X Liu Y . Comprehensive analysis of the expression and prognostic significance of the CENP family in breast cancer. Int J Gen Med. (2022) 15:3471–82. doi: 10.2147/IJGM.S354200, PMID: 35378917 PMC8976518

[20] Hao X Qiu Y Cao L Yang X Zhou D Liu J . Over-expression of centromere protein U participates in the Malignant neoplastic progression of breast cancer. Front Oncol. (2021) 11:615427. doi: 10.3389/fonc.2021.615427, PMID: 33833984 PMC8021899

[21] Ding Y Li Y Duan Y Wang W Zheng W Cheng W . LncRNA MBNL1-AS1 represses proliferation and cancer stem-like properties of breast cancer through MBNL1-AS1/ZFP36/CENPA axis. J Oncol. (2022) 2022:9999343. doi: 10.1155/2022/9999343, PMID: 35518784 PMC9064507

[22] Zeitlin SG Baker NM Chapados BR Soutoglou E Wang JY Berns MW . Double-strand DNA breaks recruit the centromeric histone CENP-A. Proc Natl Acad Sci U.S.A. (2009) 106:15762–7. doi: 10.1073/pnas.0908233106, PMID: 19717431 PMC2747192

[23] Boehm KM El Nahhas OSM Marra A Waters M Jee J Braunstein L . Multimodal histopathologic models stratify hormone receptor-positive early breast cancer. Nat Commun. (2025) 16:2106. doi: 10.1038/s41467-025-57283-x, PMID: 40025017 PMC11873197

[24] Chen L Dhoomun DK Liu Q Kong X Li X Peng S . A prognostic model based on CLEC6A predicts clinical outcome of breast cancer patients. Int Immunopharmacol. (2024) 137:112411. doi: 10.1016/j.intimp.2024.112411, PMID: 38852520

[25] Berton Giachetti PPM Carnevale Schianca A Trapani D Marra A Toss A Marchio C . Current controversies in the use of Oncotype DX in early breast cancer. Cancer Treat Rev. (2025) 135:102887. doi: 10.1016/j.ctrv.2025.102887, PMID: 40048856

[26] Rios-Hoyo A Xiong K Dai J Yau C Marczyk M Garcia-Milian R . Hormone receptor-positive HER2-negative/mammaPrint high-2 breast cancers closely resemble triple-negative breast cancers. Clin Cancer Res. (2025) 31:403–13. doi: 10.1158/1078-0432.CCR-24-1553, PMID: 39561272 PMC11747811

[27] Feng Z Chen Y Cai C Tan J Liu P Chen Y . Pan-cancer and single-cell analysis reveals CENPL as a cancer prognosis and immune infiltration-related biomarker. Front Immunol. (2022) 13:916594. doi: 10.3389/fimmu.2022.916594, PMID: 35844598 PMC9279617

[28] Kang Z Li R Liu C Dong X Hu Y Xu L . m(6)A-modified cenRNA stabilizes CENPA to ensure centromere integrity in cancer cells. Cell. (2024) 187:6035–6054.e27. doi: 10.1016/j.cell.2024.08.040, PMID: 39305902

[29] Li Q Tang Y Zuo JB Han H Tu GX Chen C . CENP-H as a new prognostic biomarker for tumors: a real-world literature review. Front Oncol. (2025) 15:1521988. doi: 10.3389/fonc.2025.1521988, PMID: 40071086 PMC11893413

[30] Wu G Fan Z Li X . CENPA knockdown restrains cell progression and tumor growth in breast cancer by reducing PLA2R1 promoter methylation and modulating PLA2R1/HHEX axis. Cell Mol Life Sci. (2024) 81:27. doi: 10.1007/s00018-023-05063-5, PMID: 38212546 PMC11072086

[31] Bai J Wang Z Yang M Xiang J Liu Z . Disrupting CENP-N mediated SEPT9 methylation as a strategy to inhibit aerobic glycolysis and liver metastasis in colorectal cancer. Clin Exp Metastasis. (2024) 41:971–88. doi: 10.1007/s10585-024-10316-z, PMID: 39424682

[32] Li Y Cai H Yang J Xie X Pei S Wu Y . Decoding tumor heterogeneity in uveal melanoma: basement membrane genes as novel biomarkers and therapeutic targets revealed by multi-omics approaches for cancer immunotherapy. Front Pharmacol. (2023) 14:1264345. doi: 10.3389/fphar.2023.1264345, PMID: 37822877 PMC10562578

[33] U. S. P. S. T. Force Nicholson WK Silverstein M Wong JB Barry MJ Chelmow D . Screening for breast cancer: US preventive services task force recommendation statement. JAMA. (2024) 331:1918–30. doi: 10.1001/jama.2024.5534, PMID: 38687503

[34] Morganti S Marra A De Angelis C Toss A Licata L Giugliano F . PARP inhibitors for breast cancer treatment: A review. JAMA Oncol. (2024) 10:658–70. doi: 10.1001/jamaoncol.2023.7322, PMID: 38512229

[35] Zhang H Zhang L He Y Jiang D Sun J Luo Q . PI3K PROTAC overcomes the lapatinib resistance in PIK3CA-mutant HER2 positive breast cancer. Cancer Lett. (2024) 598:217112. doi: 10.1016/j.canlet.2024.217112, PMID: 38986734

[36] Kundu M Butti R Panda VK Malhotra D Das S Mitra T . Modulation of the tumor microenvironment and mechanism of immunotherapy-based drug resistance in breast cancer. Mol Cancer. (2024) 23:92. doi: 10.1186/s12943-024-01990-4, PMID: 38715072 PMC11075356

[37] Lu X Gou Z Chen H Li L Chen F Bao C . Extracellular matrix cancer-associated fibroblasts promote stromal fibrosis and immune exclusion in triple-negative breast cancer. J Pathol. (2025) 265:385–99. doi: 10.1002/path.6395, PMID: 39846260

[38] Corso G Fusco N Guerini-Rocco E Leonardi MC Criscitiello C Zagami P . Invasive lobular breast cancer: Focus on prevention, genetics, diagnosis, and treatment. Semin Oncol. (2024) 51:106–22. doi: 10.1053/j.seminoncol.2024.05.001, PMID: 38897820

[39] Li YW Dai LJ Wu XR Zhao S Xu YZ Jin X . Molecular characterization and classification of HER2-positive breast cancer inform tailored therapeutic strategies. Cancer Res. (2024) 84:3669–83. doi: 10.1158/0008-5472.CAN-23-4066, PMID: 39186675

[40] Chaib M Sipe LM Yarbro JR Bohm MS Counts BR Tanveer U . PKC agonism restricts innate immune suppression, promotes antigen cross-presentation and synergizes with agonistic CD40 antibody therapy to activate CD8(+) T cells in breast cancer. Cancer Lett. (2022) 531:98–108. doi: 10.1016/j.canlet.2022.01.017, PMID: 35074498 PMC9867936

[41] Baek S Cui K . Targeting CD200 in breast cancer: opportunities and challenges in immunotherapeutic strategies. Int J Mol Sci. (2024) 26:1. doi: 10.3390/ijms26010115, PMID: 39795972 PMC11719565

[42] de Mingo Pulido A Gardner A Hiebler S Soliman H Rugo HS Krummel MF . TIM-3 regulates CD103(+) dendritic cell function and response to chemotherapy in breast cancer. Cancer Cell. (2018) 33:60–74.e6. doi: 10.1016/j.ccell.2017.11.019, PMID: 29316433 PMC5764109

[43] Joseph C Alsaleem MA Toss MS Kariri YA Althobiti M Alsaeed S . The ITIM-containing receptor: leukocyte-associated immunoglobulin-like receptor-1 (LAIR-1) modulates immune response and confers poor prognosis in invasive breast carcinoma. Cancers (Basel). (2020). 13(1):80. doi: 10.3390/cancers13010080, PMID: 33396670 PMC7795350

[44] Liu J Pei S Zhang P Jiang K Luo B Hou Z . Liquid-liquid phase separation throws novel insights into treatment strategies for skin cutaneous melanoma. BMC Cancer. (2023) 23:388. doi: 10.1186/s12885-023-10847-w, PMID: 37127623 PMC10150491

[45] Li Z Pei S Wang Y Zhang G Lin H Dong S . Advancing predictive markers in lung adenocarcinoma: A machine learning-based immunotherapy prognostic prediction signature. Environ Toxicol. (2024) 39:4581–93. doi: 10.1002/tox.24284, PMID: 38591820

[46] Lim JP Nair S Shyamasundar S Chua PJ Muniasamy U Matsumoto K . Silencing Y-box binding protein-1 inhibits triple-negative breast cancer cell invasiveness via regulation of MMP1 and beta-catenin expression. Cancer Lett. (2019) 452:119–31. doi: 10.1016/j.canlet.2019.03.014, PMID: 30905819

[47] Song Y Lu M Feng L Chen Q Huang H Lin Q . Identification of potential immunotherapy biomarkers for breast cancer by bioinformatics analysis. Biosci Rep. (2022) 42:2. doi: 10.1042/BSR20212035, PMID: 35037689 PMC8819662

[48] Si W Xu X Wan L Lv F Wei W Xu X . RUNX2 facilitates aggressiveness and chemoresistance of triple negative breast cancer cells via activating MMP1. Front Oncol. (2022) 12:996080. doi: 10.3389/fonc.2022.996080, PMID: 36483054 PMC9724742

[49] Akter T Aziz MA Islam MS Sarwar MS . Association of MMP1 gene polymorphisms with breast cancer risk: A narrative review. Health Sci Rep. (2023) 6:e1607. doi: 10.1002/hsr2.1607, PMID: 37841939 PMC10570771

[50] Yu ZH Xu HL Wang S Li YX Wang GX Tian Y . Integrating spatial and single-cell transcriptomes reveals the role of COL1A2(+) MMP1(+/-) cancer-associated fibroblasts in ER-positive breast cancer. Cancer Cell Int. (2025) 25:82. doi: 10.1186/s12935-025-03705-1, PMID: 40055751 PMC11887395

[51] Eiro N Cid S Aguado N Fraile M de Pablo N Fernandez B . MMP1 and MMP11 expression in peripheral blood mononuclear cells upon their interaction with breast cancer cells and fibroblasts. Int J Mol Sci. (2020) 22(1):371. doi: 10.3390/ijms22010371, PMID: 33396463 PMC7795480

[52] Kim HW Park JE Baek M Kim H Ji HW Yun SH . Matrix metalloproteinase-1 (MMP1) upregulation through promoter hypomethylation enhances tamoxifen resistance in breast cancer. Cancers (Basel). (2022) 14(5):1232. doi: 10.3390/cancers14051232, PMID: 35267540 PMC8909089

