# Supplementary material for: Advancing breast cancer biomarkers: a centromere-related gene signature integrated with single-cell analysis for prognostic prediction
Source: Front Immunol. 2025 Dec 4;16:1678603. doi: 10.3389/fimmu.2025.1678603 (PMC12711834; doi:10.3389/fimmu.2025.1678603)
Supplement: Supplementary file 1 [file Table1.docx]

| **Oligonucleotides** | **Nucleotide sequence (5'-3')** |
| --- | --- |
| **siRNA** |  |
| Scramble control | GCUUCGCGCCGUAGUCUUA |
| Si-MMP1-1 | TGAAGATGAAAGGTGGACCAA |
| Si-MMP1-2 | GTTTGTGGCTTATGGATTCAT |
|  |  |
| **Primer** |  |
| GAPDH | GGCCTCCAAGGAGTAAGACC (forward) |
|  | AGGGGAGATTCAGTGTGGTG (reverse) |
| MMP1 | AGAAAGAAGACAAAGGCAAGTTGA (forward) |
|  | GCATGGTCCACATCTGCTCT (reverse) |
|  |  |

**Supplementary Table 1. Oligonucleotides used in research**
